# Associations of traditional social support and *dazi* socializing with social anxiety in medical students: the mediating role of psychological resilience

**DOI:** 10.3389/fpsyg.2026.1894684

**Published:** 2026-09-24

**Authors:** Le Shi, Liangliang Chen, Xiang Chen, Jialin Xie, Xinlu Lin

**Affiliations:** The Second Clinical Medical College, Zhejiang Chinese Medical University, Hangzhou, China

**Keywords:** traditional social support, *dazi* socializing, medical students, psychological resilience, social anxiety, mediation analysis

## Abstract

**Objective:**

This study investigated associations of traditional social support (family and friend support) and *dazi* socializing with social anxiety among medical students, and examined the mediating role of psychological resilience, addressing gaps in integrating the stress-buffering model and conservation of resources theory.

**Methods:**

Convenience sampling was used to recruit undergraduate students majoring in clinical medicine from the first to fifth years at a university in Zhejiang Province. Participants completed online questionnaires including the Self-developed Questionnaire on Experiences of *dazi* Socializing, Chinese Version of the Perceived Social Support Scale, Interaction Anxiousness Scale and the Connor-Davidson Resilience Scale. Data cleaning and statistical analyses were performed using Python 3.11. ANCOVA was conducted to control for gender, academic year, and student leadership. The mediating effect of psychological resilience was examined using model 4 of Hayes's PROCESS macro and Bootstrap method based on 5,000 resampling iterations. Pearson correlation, hierarchical regression, and independent-samples t tests were applied for intra-group analyses among participants with *dazi* experiences.

**Results:**

After controlling for covariates, the *dazi* group scored higher than non-*dazi* users in friend support (*F*_(1,677)_ = 27.18, *p* < 0.001), family support (*F*_(1,677)_ = 8.45, *p* = 0.004), and psychological resilience (*F*_(1,677)_ = 18.80, *p* < 0.001). Meanwhile, the *dazi* group reported lower social anxiety than non-*dazi* users (*F*_(1,677)_ = 22.23, *p* < 0.001). Mediation analysis showed that psychological resilience fully mediated friend support (indirect effect = −0.0211, 95% CI [−0.0433, −0.0021], 23.4%) and family support (indirect effect = −0.0159, 95% CI [−0.0340, −0.0017], 33.1%) on social anxiety. It partially mediated *dazi* socializing (direct effect = −1.2215, 95% CI [−1.8773, −0.5439]; indirect effect = −0.0812, 95% CI [−0.1951, −0.0033], 6.2%). Within the *dazi* group, higher social anxiety was indirectly associated with greater “Safety/Trust concerns”; emotional *dazi* scored lower on resilience than functional *dazi*, with the difference approaching significance (*t* = −1.94, *p* = 0.054).

**Conclusion:**

Both traditional social support and *dazi* experiences are associated with social anxiety levels among medical students. The relationship between traditional social support and social anxiety is entirely indirect through psychological resilience, whereas the association between *dazi* experiences and social anxiety is achieved through both direct and indirect pathways via psychological resilience.

## 1 Introduction

Social support refers to external resources that individuals obtain from social relationships and that can buffer the negative effects of stress and promote physical and mental health (Cobb, 1976; Cohen and Wills, 1985; Thoits, 2011). Traditional social support is rooted in the differential mode of association bound by blood and geographic ties, with its core characteristics being high relational strength and emotional depth. Individuals are embedded within social networks centered on the family and extending outward in layers according to relational proximity, and this proximity determines the density of resource allocation and emotional connections. Within this framework, strong ties constitute the primary vehicle of social support (Fei, 2022). Granovetter (1973) defined tie strength based on the frequency of interaction, emotional intensity, intimacy, and reciprocal exchange, positing that strong ties are predominantly found among family members and close friends, characterized by high information homogeneity and deep emotional investment (Granovetter, 1973). Therefore, traditional social support is essentially a support system built upon strong ties, regulated by differential relational ethics, and sustained through emotional reciprocity and long-term obligations.

*Dazi* socializing refers to a form of social engagement in which users find convenient, comfortable, and highly compatible companionship through precisely targeted interactions within niche domains based on shared interests or specific needs (Wang, 2024; Qian et al., 2007). Its prevalence is closely associated with macrosocial factors, shifts in young people's social attitudes, and technological developments (Wang and Hu, 2023). As contemporary youth transition from an acquaintance-based society to a stranger society, social interactions have increasingly emphasized personal boundaries, making *dazi* socializing a rational choice (Liu, 2023). Compared with traditional strong-tie relationships, *dazi* relationships are centered on temporary companionship within specific contexts, involving relatively limited emotional investment. Participants in such relationships “seek what they need and come and go freely,” without the need to devote substantial effort to maintaining these connections (Zhou et al., 2026). These characteristics correspond to the features of weak ties, including low interaction frequency, low emotional intensity, and limited reciprocal obligations (Granovetter, 1973; Li and Feng, 2025), which play a unique role in information diffusion, resource acquisition, and social support provision, and might significantly contribute to individuals' subjective well-being and sense of belonging (Sandstrom and Dunn, 2014).

University students are at a critical stage of transitioning from family attachment to peer reliance, a period in which traditional strong ties may weaken due to spatial separation and academic pressures (Parade et al., 2010); Medical students, in particular, face the dual challenges of continuously changing social circles and disruptions in deep emotional support due to prolonged educational trajectories and frequent clinical rotations (Koo et al., 2025). Within traditional social support systems, family support provides individuals with emotional belonging and a sense of security, whereas friend support satisfies individuals' needs for belonging and identity recognition. However, traditional social support systems rely heavily on physical co-presence and the maintenance of long-term relationships, and their supportive functions are constrained by multiple factors, including geographical distance, time costs, and relationship depth (Chen, 2015). As an emerging weak-tie social support system, *dazi* socializing partially fills the gaps left by traditional social support. Specifically, it provides functional support (e.g., study partners facilitating the sharing of learning materials), situational support (e.g., meal partners providing reminders for maintaining regular routines), and identity-related support (e.g., postgraduate entrance examination partners strengthening professional identity) (Zhang et al., 2024). Nevertheless, the temporary and boundary-oriented characteristics of *dazi* socializing may result in superficial emotional support and may also impede the effective transformation of external resources into psychological resilience (Lian and Liu, 2024).

Social anxiety refers to emotional responses characterized by tension, fear, or discomfort that individuals experience in social or performance situations due to anticipated negative evaluations from others (Leary, 1983; Rapee and Heimberg, 1997). Its adverse effects on medical students are multidimensional and profound. Regarding core clinical competencies, it is significantly negatively associated with physician-patient communication behaviors, indirectly impairing communication effectiveness by reducing social self-esteem (Guo et al., 2015). In terms of career orientation, it significantly influences specialty choice, resulting in an “avoidance-choice” bias that may contribute to structural imbalances in the allocation of medical professionals (Cohen and Wills, 1985). From the perspective of the stress-buffering model, social anxiety can be understood as an individual's stress response to anticipated social evaluation threats, whereas social support serves as a critical resource for buffering such responses (Abudasser et al., 2025; Mohamed et al., 2026).

Psychological resilience refers to individuals' capacity for positive adaptation and rapid recovery in response to adversity, trauma, or significant stress through the dynamic interaction of internal and external protective resources (Connor and Davidson, 2003; Masten, 2001; Ozbay et al., 2008). It is not only associated with the lower resource loss but may also facilitate resource gain. The process through which social support (external resources) is linked with lower anxiety via higher psychological resilience (internal resources) exemplifies a prototypical resource conversion process (Hobfoll, 1989; Thompson et al., 2016). Examining how psychological resilience mediates the effects of traditional social support and *dazi* experience on social anxiety can provide intervention targets for mental health education among medical students.

Existing studies have explored the relationships among social support, psychological resilience, and social anxiety. However, these studies have primarily focused on general university student populations, while research targeting medical students, a population characterized by high stress levels and substantial social demands, remains relatively limited. Furthermore, *dazi* socializing, as a novel form of weak-tie support, has not yet been incorporated into mainstream social support research frameworks. The functional differences between *dazi* socializing and traditional strong-tie support, as well as its unique pathways influencing psychological health, remain unclear. Accordingly, this study proposes the following hypotheses: H1: Traditional social support (friend support and family support) and *dazi* socializing experiences are negatively associated with social anxiety among medical students. H2: Traditional social support (friend support and family support) and *dazi* socializing experiences are positively associated with psychological resilience among medical students. H3: Psychological resilience is negatively associated with social anxiety among medical students. H4: Psychological resilience is associated with both traditional social support and social anxiety, and it is associated with both *dazi* socializing experiences and social anxiety.

## 2 Methods

### 2.1 Participants

A convenience sampling method was employed to recruit undergraduate students majoring in clinical medicine from the first to fifth years at a university in Zhejiang Province. Before data collection, participants were informed of the study objectives, procedures, principles of anonymity and confidentiality, as well as their right to participate voluntarily or withdraw at any time. Questionnaires were distributed online, yielding 750 completed responses, with a response rate of 86.31%. Inclusion criteria were: (1) full-time enrolled undergraduates majoring in clinical medicine; (2) provided informed consent and participated voluntarily. Exclusion criteria were: (1) a history of diagnosed severe psychiatric illness within the past six months; (2) currently taking psychotropic medications; (3) questionnaire completion time < 120 seconds or > 1800 seconds; (4) responses exhibiting obvious regularities (e.g., selecting the same option for more than 10 consecutive items); (5) duplicate submissions from the same IP address, where only the first response was retained. Ultimately, 685 valid questionnaires were obtained, yielding a valid response rate of 91.33%.

In this study, all variables had complete data with no missing values, except for the self-developed questionnaire on *dazi* socializing experiences. This questionnaire was administered only to participants who had such experiences and was therefore not applicable to those without them.

In terms of sample-size adequacy, a priori power analysis was performed using G Power 3.1. The significance level was set at α = 0.05 (two-tailed), and statistical power 1 - β = 0.80. Conservative small-to-medium effect sizes were adopted to determine the minimum sample size required to achieve adequate statistical power under expected effect sizes, based on which the data-collection scale was planned. Effect-size benchmarks followed (Cohen, 1992). Since G Power does not support direct *a priori* calculation for bootstrap-based mediation models, approximate estimation was conducted within the multiple-regression framework following Fritz and MacKinnon (2007), and the resultant sample size was inflated by 30 % to compensate for losses induced by bootstrap resampling.

1. Independent-samples *t*-test. Used for group comparisons between participants with versus without *dazi* socializing experiences, and across groups with different *dazi* socializing motive types. To detect a small effect (*d* = 0.20), the required total sample size was *N* = 398; to detect a medium effect (*d* = 0.50), the required total sample size was *N* = 128.
2. Analysis of covariance (ANCOVA). A binary independent variable was specified, with gender, academic year, and student leadership status included as three covariates. To detect a small effect (*f* = 0.10), the required total sample size was *N* = 313; to detect a medium effect (*f* = 0.25), the required total sample size was *N* = 60.
3. Mediation-effect analysis. Three demographic variables were controlled. After 30 % sample-size inflation for correction, the required sample size was *N* = 212 for small effects and *N* = 57 for medium effects.
4. Hierarchical regression analysis. This analysis examined the incremental predictive power of *dazi* socializing experiences on social anxiety. The full model contained five predictors, with three additional predictors entered relative to the baseline model. To detect a small effect (*f*^2^ = 0.02), the required total sample size was *N* = 207; to detect a medium effect (*f*^2^ = 0.15), the required total sample size was *N* = 47.

In summary, 685 valid questionnaires were finally obtained in this study (*n* = 362 for the *dazi* group, *n* = 323 for the non-*dazi* group), which guaranteed sufficient statistical power even when detecting small-to-medium effect sizes.

### 2.2 Measurement instruments

#### 2.2.1 Self-developed basic information questionnaire

A self-developed basic information questionnaire was used to collect sociodemographic information and experiences of *dazi* socializing among all participants. The sociodemographic information included gender, academic year, place of origin, single-child status, and student leadership status. *Dazi* socializing experiences included: whether participants were aware of *dazi* socializing, and whether they had experiences of seeking or having *dazi*.

#### 2.2.2 Self-developed questionnaire on experiences of *dazi* socializing

Participants who selected “yes” for the item “whether they were aware of *dazi* socializing” in the self-developed basic information questionnaire were directed via the platform's skip logic to complete the self-developed questionnaire on *dazi* socializing experiences. This questionnaire comprised the following four dimensions:

1. Cognition and attitudes: ① Core differences between *dazi* and traditional friends (multiple-choice) ② Overall attitude toward *dazi* socializing;
2. Motivational needs: Needs satisfied by *dazi* socializing (multiple-choice question);
3. Behavioral characteristics: ① Channels for seeking *dazi* (multiple-choice question) ② Types of *dazi* (multiple-choice question) ③ Frequency of interactions with *dazi*;
4. Experience evaluation.

#### 2.2.3 Chinese version of the perceived social support scale (PSSS)

Social support was assessed across all participants using the Chinese version of Perceived Social Support Scale revised by Jiang Qianjin (Jiang, 2001). The scale consists of 12 items and includes three dimensions: family support, friend support, and other support. This study only adopted dimensions of family support and friend support (8 items in total), rated on a 7-point Likert scale (1 = strongly disagree, 7 = strongly agree), with total scores ranging from 8 to 56. Higher scores indicate higher levels of perceived social support in the corresponding dimension. In this study, the Cronbach's α coefficients for the family support and friend support dimensions were 0.908 and 0.901, respectively.

#### 2.2.4 Chinese version of the interaction anxiousness scale (IAS)

All participants completed the Chinese version of the Interaction Anxiousness Scale (IAS). The revised Chinese version adapted by Peng et al. (2004) was adopted, consisting of 15 items that measure individuals' subjective anxiety experiences during interpersonal interactions. Responses were rated on a 5-point Likert scale (1 = not at all characteristic of me, 5 = extremely characteristic of me). Items 3, 6, 10, and 15 were reverse-scored. Total scores ranged from 15 to 75, with higher scores indicating more severe social anxiety symptoms. The Cronbach's α coefficient of this scale in this study was 0.766.

#### 2.2.5 Chinese version of the Connor-Davidson resilience scale (CD-RISC)

All participants completed the Chinese version of the Connor-Davidson Resilience Scale (CD-RISC) (Yu and Zhang, 2007). The revised Chinese version developed by Yu Yixing et al. was used. The scale contains 25 items and assesses individuals' adaptive capacity under stressful conditions. The total score ranges from 25 to 125. Scores of 86-94 indicate normal levels of psychological resilience, scores of 95-104 indicate mild levels, and scores of ≥105 indicate excellent levels of psychological resilience. Higher scores indicate better psychological resilience. The Cronbach's α coefficient of this scale in this study was 0.933.

### 2.3 Statistical methods

Data cleaning and statistical analyses were performed using Python 3.11 (Statsmodels 0.14.0, Scipy 1.11.0). All statistical tests were two-tailed, and *p* < 0.05 was considered statistically significant. Categorical variables were presented as frequencies (percentages), whereas continuous variables were presented as means ± standard deviations (M ± SD). Between-group comparisons of categorical variables were conducted using Pearson's chi-square test (χ^2^ test), and Fisher's exact test was applied when any expected frequency was less than 5. Comparisons of continuous variables between two groups were conducted using independent-samples t tests with Welch correction, without assuming equal variances. Effect sizes were reported using Cohen's *d*, with *d* = 0.2, 0.5, and 0.8 representing small, medium, and large effects, respectively. Given that demographic variables (gender, academic year, and student leadership status) showed significant differences in distribution between groups, analysis of covariance (ANCOVA) was conducted by including these variables as covariates to control for their potential confounding effects on the relationships among core variables.

The mediating effect of psychological resilience was tested using Model 4 of the PROCESS macro (Bootstrap method) proposed by Hayes (2013). Gender, academic year, and student leadership status were included as control variables; friend support scores, family support scores, and *dazi* socializing experiences (with or without experience) were included as independent variables; psychological resilience was included as the mediator; and social anxiety was included as the dependent variable. Bootstrap resampling was conducted 5,000 times, and an effect was considered significant when the 95% bias-corrected percentile Bootstrap confidence interval (95% CI) did not include zero. The proportion of the mediation effect was calculated using the following formula:


$$\begin{array}{l} \text{The proportion of the mediation effect }=\;\frac{\text{indirect effect}}{\text{total effect}}\;\times 100\%. \end{array}$$

Furthermore, in-depth intra-group analyses were conducted among participants with *dazi* socializing experiences: (1) Pearson correlation analysis was conducted to examine the associations between *dazi* experience variables (sense of ease, Safety/Trust concerns, and barriers to deep relationships) and traditional support as well as psychological outcome; (2) Hierarchical regression analysis was performed to evaluate the incremental predictive power of *dazi* experiences on social anxiety: Model 1 included traditional support (friend support and family support), whereas Model 2 additionally incorporated *dazi* experience indicators (*dazi* enjoyment, *dazi* ease, and Safety/Trust concerns). The values of *R*^2^ and Δ*R*^2^ were reported; (3) Independent-samples *t* tests with Welch correction were conducted to compare differences in psychological resilience and social anxiety between different types of *dazi* motivations (emotional-oriented vs. function-oriented). For the regression models (mediation analysis and hierarchical regression), multicollinearity was assessed using variance inflation factors (VIFs), with VIF > 10 indicating potential collinearity; homoscedasticity was evaluated using the Breusch–Pagan test; and residual normality was examined using the Shapiro–Wilk test with Q–Q plots for visual inspection.

## 3 Results

### 3.1 Basic characteristics of participants

A total of 685 valid participants were included in this study: 362 (52.8%) with *dazi* socializing experiences and 323 (47.2%) without. The results of the chi-square tests (see Table 1) indicated statistically significant differences between the two groups in gender distribution (χ^2^ = 92.28, *p* < 0.001), academic year distribution (χ^2^ = 22.49, *p* < 0.001), and student leadership status (χ^2^ = 6.20, *p* = 0.013).

**Table 1:** Comparison of demographic characteristics between medical students with and without dazi experiences [*n* (%)].

Variable	Without *dazi* experience group (*n* = 323)	With *dazi* experience group (*n* = 362)	χ^2^	*P*-value
Gender			92.28	< 0.001
Male	165 (51.1%)	59 (16.3%)		
Female	158 (48.9%)	303 (83.7%)		
Academic year			22.49	< 0.001
First year	137 (42.4%)	177 (48.9%)		
Second year	58 (18.0%)	76 (21.0%)		
Third year	48 (14.9%)	62 (17.1%)		
Fourth year	52 (16.1%)	19 (5.2%)		
Fifth year	28 (8.7%)	28 (7.7%)		
Place of origin			2.51	0.285
Urban area	110 (34.1%)	144 (39.8%)		
County/township area	109 (33.7%)	108 (29.8%)		
Rural area	104 (32.2%)	110 (30.4%)		
Single-child status			0.24	0.623
Yes	121 (37.5%)	128 (35.4%)		
No	202 (62.5%)	234 (64.6%)		
Student leadership status			6.20	0.013
Yes	116 (35.9%)	165 (45.6%)		
No	207 (64.1%)	197 (54.4%)		

Data are presented as frequencies (percentages). The p-values were calculated based on chi-square tests (χ^2^ tests) to examine distributional differences in demographic variables between the two groups. A *p*-value < 0.05 indicates a statistically significant difference.

Specifically, the proportion of female participants (83.7%) and student leaders (45.6%) was significantly higher in the *dazi* group than in the non-*dazi* group (48.9% and 35.9%, respectively). Women generally exhibit stronger relationship orientation and greater social initiative (Drescher and Schultheiss, 2016), which may make them more inclined to use *dazi* relationships, a low-threshold and flexible form of social interaction, to fulfill diverse social needs. Student leaders typically participate in more collective activities and organizational coordination tasks, and their social opportunities and needs are inherently greater, providing them with more opportunities to establish *dazi* relationships.

Regarding academic year distribution, the *dazi* group was primarily composed of first-year students (48.9%), whereas the non-*dazi* group showed a relatively balanced distribution across academic years. This finding suggests that *dazi* socializing may be an important strategy for first-year students to adapt to a new environment and establish new social networks.

No statistically significant differences were observed between the two groups in place of origin (χ^2^ = 2.51, *p* = 0.285) or single-child status (χ^2^ = 0.24, *p* = 0.623).

Given the significant imbalance in the distribution of gender, academic year, and student leadership status between groups, these variables were included as covariates for statistical control in subsequent analyses.

### 3.2 Comparison of core variables between medical students with and without *dazi* experiences

To examine the relationship between *dazi* socializing and individuals' psychological health as well as social support systems, independent-samples t tests with Welch correction were conducted to compare differences between the two groups in friend support scores, family support scores, social anxiety, and psychological resilience. Cohen's *d* was used to report effect sizes. Furthermore, analysis of covariance (ANCOVA) was conducted by including gender, academic year, and student leadership status as covariates to control for the confounding effects of demographic variables. The results are presented in Table 2.

**Table 2:** Comparison of core variables between medical students with and without *dazi* experiences (M ± SD).

Variable	Without *dazi* experience group (*n* = 323)	With *dazi* experience group (*n* = 362)	*t*-value	Cohen's *d*	ANCOVA (a)
Friend support total score	20.30 ± 4.29	21.83 ± 3.97	4.84^***^	0.37	27.18^***^
Family support total score	20.61 ± 4.85	21.18 ± 4.51	1.59	0.12	8.45^**^
Social anxiety	48.15 ± 3.88	46.86 ± 3.94	−4.31^***^	−0.33	22.23^***^
Psychological resilience	83.73 ± 12.52	86.68 ± 12.34	3.09^**^	0.24	18.80^***^

^**^ p < 0.01.

^***^ p < 0.001.

The *t* values were based on Welch's *t* tests. (a) ANCOVA was conducted with gender, academic year, and student leadership status as covariates.

The independent-samples t test results (Welch correction) showed that the *dazi* experience group had significantly higher friend support scores than the non-*dazi* experience group (*t*_(659)_ = 4.84, *p* < 0.001, *d* = 0.37). The *dazi* experience group also demonstrated significantly higher levels of psychological resilience than the non-*dazi* experience group (*t*_(672)_ = 3.09, *p* = 0.002, *d* = 0.24). In contrast, the *dazi* experience group reported significantly lower social anxiety scores than the non-*dazi* experience group (*t*_(676)_ = −4.31, *p* < 0.001, *d* = −0.33). No statistically significant difference was observed between the two groups in family support scores (*t*_(660)_ = 1.59, *p* = 0.113, *d* = 0.12). These findings suggest that *dazi* socializing is not an “ineffective form of social interaction” but a meaningful supplement to the social support system in campus contexts.

After controlling for gender, academic year, and student leadership status, ANCOVA results indicated that the between-group differences in friend support (*F*_(1, 677)_ = 27.18, *p* < 0.001), family support (*F*_(1, 677)_ = 8.45, *p* = 0.004), social anxiety (*F*_(1, 677)_ = 22.23, *p* < 0.001), and psychological resilience (*F*_(1, 677)_ = 18.80, *p* < 0.001) remained statistically significant. Notably, the difference in family support, which was initially non-significant, became significant after controlling for covariates (*p* = 0.004). This indicates that demographic variables may have produced a suppression effect in this relationship (MacKinnon et al., 2000). After controlling for these demographic variables, individuals with *dazi* experiences appeared to have higher levels of family support. Assumption checks for the ANCOVA models indicated that Levene's tests were non-significant for all four dependent variables (ps > 0.05), satisfying the homogeneity of variance assumption. The Shapiro–Wilk tests were significant (ps < 0.001) due to the large sample size; however, visual inspection of Q–Q plots revealed only mild deviations from normality, and ANCOVA is robust to such minor violations. The homogeneity of regression slopes assumption was satisfied for psychological resilience (*F* = 0.623, *p* = 0.712) but not for friend support (*F* = 2.393, *p* = 0.027), family support (*F* = 4.392, *p* < 0.001), or social anxiety (*F* = 2.767, *p* = 0.012). This suggests that the relationships between covariates and these three outcomes differed somewhat between the *dazi* and non-*dazi* groups, consistent with the observation that *dazi* experience may moderate the associations between demographic factors and psychological outcomes.

### 3.3 Survey of *dazi* socializing experiences

To further characterize the specific patterns of *dazi* socializing among medical students, a descriptive analysis was conducted among participants with *dazi* experiences (*n* = 362). The analysis covered multiple dimensions, including cognition, attitudes, motivations, channels, types, and interaction frequency.

#### 3.3.1 Core cognition and attitudes

Regarding the core differences between *dazi* relationships and traditional friendships, the three items with the highest recognition rates were “stronger purpose orientation/functionality” (72.9%), “lower social pressure” (66.6%), and “clearer interpersonal boundaries” (62.4%). In contrast, “shallower emotional depth” (51.4%) and “shorter relationship duration” (40.1%) received relatively lower recognition rates. These findings suggest that medical students perceive *dazi* relationships as a low-burden, goal-oriented, and boundary-clear form of social interaction, while such relationships do not completely exclude emotional connections.

Regarding overall attitudes, 87.9% of participants expressed either “strong support” (27.1%) or “moderate support” (60.8%) for *dazi* socializing, whereas only 1.9% reported being “not very supportive”, and no participants expressed “strong opposition.” The mean attitude score was 3.13±0.66 (maximum score = 4), indicating an overall tendency toward positive acceptance.

#### 3.3.2 Motivations and behavioral characteristics

Among the needs fulfilled by *dazi* socializing, three items had recognition rates exceeding 70%: “functional needs (cost-sharing or making up the numbers)” (80.7%), “companionship needs (avoiding loneliness)” (76.5%), and “interest-sharing needs (finding like-minded people)” (72.1%). The next most frequently recognized needs were “security needs (companionship for safety)” (50.3%), “low-pressure social needs” (48.9%), and “efficiency needs (saving time and money)” (43.6%). These findings reveal the essence of *dazi* socializing: it exchanges relatively low emotional costs (low burden) for immediate companionship benefits, which aligns well with the high-pressure and fragmented lifestyle of medical students.

Regarding channels for finding *dazi*, “naturally meeting through offline activities” (55.8%) and “introduced by acquaintances” (52.8%) were the most common approaches. These were followed by “social media platforms (e.g., Xiaohongshu)” (49.2%) and “interest-based communities (WeChat groups/QQ groups)” (38.4%), whereas dedicated social networking applications showed a very low usage rate (5.5%). This suggests that *dazi* relationships among medical students rely heavily on offline contexts and trust transmission through familiar social networks.

In terms of *dazi* types, the most common type was “meal companions” (68.8%), followed by “study companions” (48.3%) and “gaming companions” (36.7%). “Exercise and fitness companions” had the lowest recognition rate (20.2%). This distribution is highly consistent with the daily routines of medical students: eating represents the most frequent and rigid social context, whereas academic pressure generates demand for study companionship.

Regarding interaction frequency (see Table 3), “irregular interaction” (55.8%) was overwhelmingly predominant. Among the 160 participants with a fixed interaction frequency (44.2%), the mean frequency score was 2.38 ± 1.21 (1 = several times per day, 5 = once per month), which generally fell between “once or twice per week” and “three to five times per week.” This indicates that the core characteristic of *dazi* relationships is their on-demand nature.

**Table 3:** Interaction frequency of *dazi* socializing.

Interaction frequency	*n* (%)
Several times per day	51 (14.1%)
Once per day	33 (9.1%)
3–5 times per week	50 (13.8%)
1–2 times per week	16 (4.4%)
Once per month	10 (2.8%)
Irregular interaction	202 (55.8%)
Regular interacting participants a	160 (44.2%)

“Regularly interacting participants a” refer to the total number of participants who reported the five fixed interaction frequencies, including several times per day, once per day, 3–5 times per week, 1–2 times per week, and once per month (*n* = 160). “Irregular interaction” refers to interactions that depend on specific needs and do not follow a fixed pattern.

#### 3.3.3 Association between *dazi* types and psychological resilience and social anxiety

Independent-samples t tests with Welch correction showed (see Table 4) that individuals with the three types of *dazi* relationships did not significantly differ from those without these types of *dazi* relationships in terms of psychological resilience or social anxiety (all *p* > 0.05). These findings indicate that specific types of *dazi* relationships are not significant distinguishing factors for psychological adaptation. The potential psychological protective role of *dazi* relationships may derive from the state of “having a *dazi*” itself. That is, possessing an accessible and responsive companionship network, rather than from any specific type of *dazi* relationship in a particular context.

**Table 4:** Association between *dazi* types and psychological resilience and social anxiety.

Dazi types (yes vs. no)	Psychological resilience (M ±SD)	*t*	*p*	Social anxiety (M ±SD)	*t*	*p*
Meal companions		−0.134	0.893		0.360	0.719
Yes (*n* = 249)	86.62 ± 12.44			46.92 ± 3.89		
No (*n* = 113)	86.81 ± 12.16			46.75 ± 4.05		
Study companions		1.188	0.236		1.245	0.214
Yes (*n* = 175)	87.47 ± 11.33			47.13 ± 4.10		
No (*n* = 187)	85.94 ± 13.20			46.61 ± 3.77		
Gaming companions		0.404	0.687		−0.141	0.888
Yes (*n* = 133)	87.01 ± 11.18			46.83 ± 3.74		
No (*n* = 229)	86.48 ± 12.98			46.89 ± 4.05		

### 3.4 Mediating effect of psychological resilience in the relationship between social support and social anxiety

To examine the mediating role of psychological resilience in the relationships between traditional social support (friend support and family support) as well as *dazi* experiences and social anxiety, mediation analyses were conducted using the PROCESS macro Model 4 developed by Hayes (2013). Gender, academic year, and student leadership status were included as control variables. The number of Bootstrap resamples was set to 5,000, and a 95% confidence interval (95% CI) excluding zero was used as the criterion for determining the significance of the mediation effect. The results are presented in Table 5 and Figure 1.

**Table 5:** Mediation effects of psychological resilience.

Independent variable	Indirect effect (a × b)	Boot SE	Boot 95% CI	Direct effect (c')	Total effect (c)	Proportion
Friend support total score	−0.0211	0.0105	[−0.0433, −0.0021]	−0.0685	−0.0900	23.4%
Family support total score	−0.0159	0.0082	[−0.0340, −0.0017]	−0.0320	−0.0480	33.1%
Dazi socializing	−0.0812	0.0487	[−0.1951, −0.0033]	−1.2215	−1.3047	6.2%

Bootstrap resampling was conducted with 5,000 iterations; CI represents the bias-corrected percentile Bootstrap confidence interval; all analyses controlled for gender, academic year, and student leadership status.

**Figure 1:**
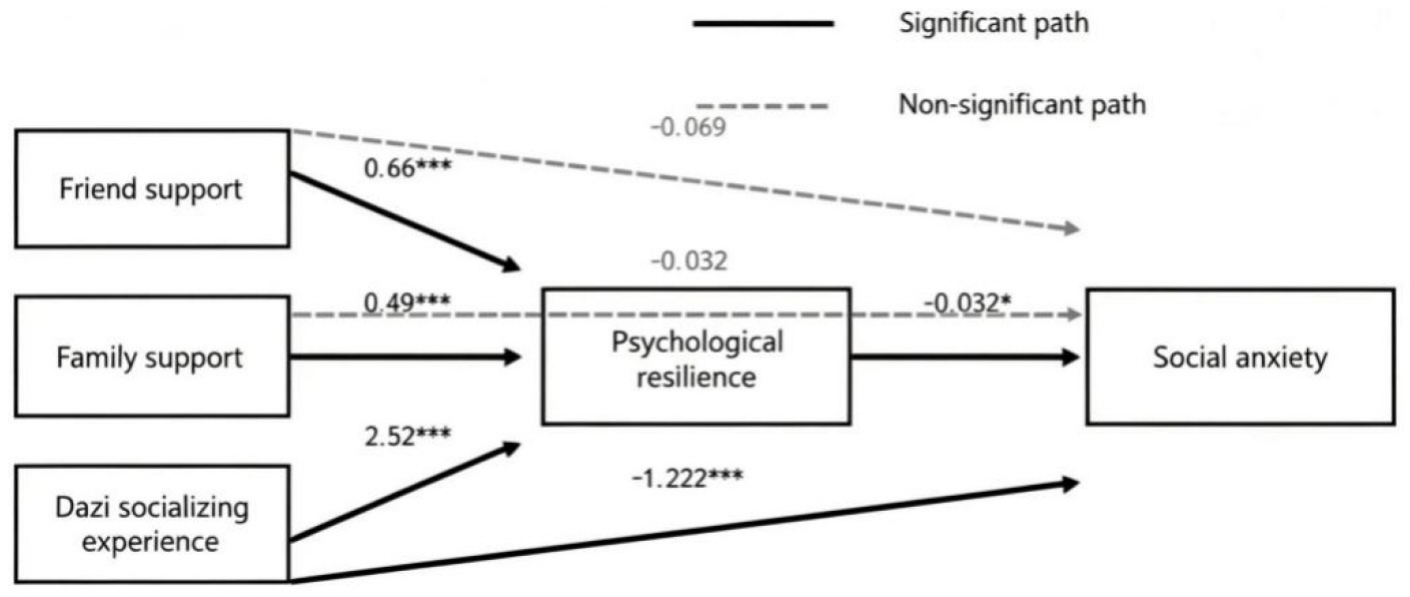
Mediation model of psychological resilience. Coefficients are unstandardized regression coefficients; ***p < 0.001, *p < 0.05; dashed arrows indicate complete mediation paths (direct effects are not significant); all analyses controlled for gender, academic year, and student leadership status.

#### 3.4.1 Friend support → psychological resilience → social anxiety

The total effect of friend support on social anxiety was significant (*c* = −0.0900, 95% CI [-0.1822,−0.0037]). After psychological resilience was included in the model, the direct effect of friend support on social anxiety became non-significant (*c'* = −0.0685, 95% CI [−0.1592, 0.0140]), whereas the indirect effect through psychological resilience was significant (a × b = −0.0211, 95% CI [−0.0433, −0.0021]). The mediating effect accounted for 23.4% of the total effect. This means that the relationship between friend support and social anxiety is entirely indirect through psychological resilience.

#### 3.4.2 Family support → psychological resilience → social anxiety

The total effect of family support on social anxiety was not significant (*c* = −0.0480, 95% CI [−0.1242, 0.0278]). After psychological resilience was included in the model, the direct effect of family support on social anxiety remained non-significant (*c'* = −0.0320, 95% CI [−0.1074, 0.0421]). However, the indirect effect through psychological resilience was significant (a × b = −0.0159, 95% CI [−0.0340, −0.0017]), accounting for 33.1% of the total effect. This means that the relationship between family support and social anxiety is entirely indirect through psychological resilience (Hayes, 2013; Baron and Kenny, 1986).

#### 3.4.3 *Dazi* socializing → psychological resilience → social anxiety

The total effect of *dazi* socializing on social anxiety was significant (*c* = −1.3047, 95% CI [−1.9503, −0.6342]). After psychological resilience was included in the model, the direct effect of *dazi* socializing on social anxiety remained significant (*c'* = −1.2215, 95% CI [−1.8773, −0.5439]). The indirect effect through psychological resilience was also significant (a × b = −0.0812, 95% CI [−0.1951, −0.0033]), accounting for 6.2% of the total effect. This means that the relationship between *dazi* experience and social anxiety involves both a direct association and an indirect association through psychological resilience.

### 3.5 Within the *dazi* group: differential effects of *dazi* experience quality

To further investigate how variations in the quality of *dazi* socializing influence psychological well-being, correlation analyses, hierarchical regression analyses, and motivation-based group comparisons were conducted within the group with *dazi* experiences (*n* = 362).

#### 3.5.1 Correlation analysis of core variables

Pearson correlation analysis showed (see Figure 2) that friend support (*r* = 0.25, *p* < 0.001) and family support (*r* = 0.28, *p* < 0.001) were significantly positively correlated with psychological resilience and significantly negatively correlated with social anxiety (*r* = −0.10~-0.18, *p* < 0.05), which was consistent with the findings from the full sample analysis. This finding suggests that individuals with stronger traditional support (friend support and family support) are also more likely to obtain a sense of ease from *dazi* relationships.

**Figure 2:**
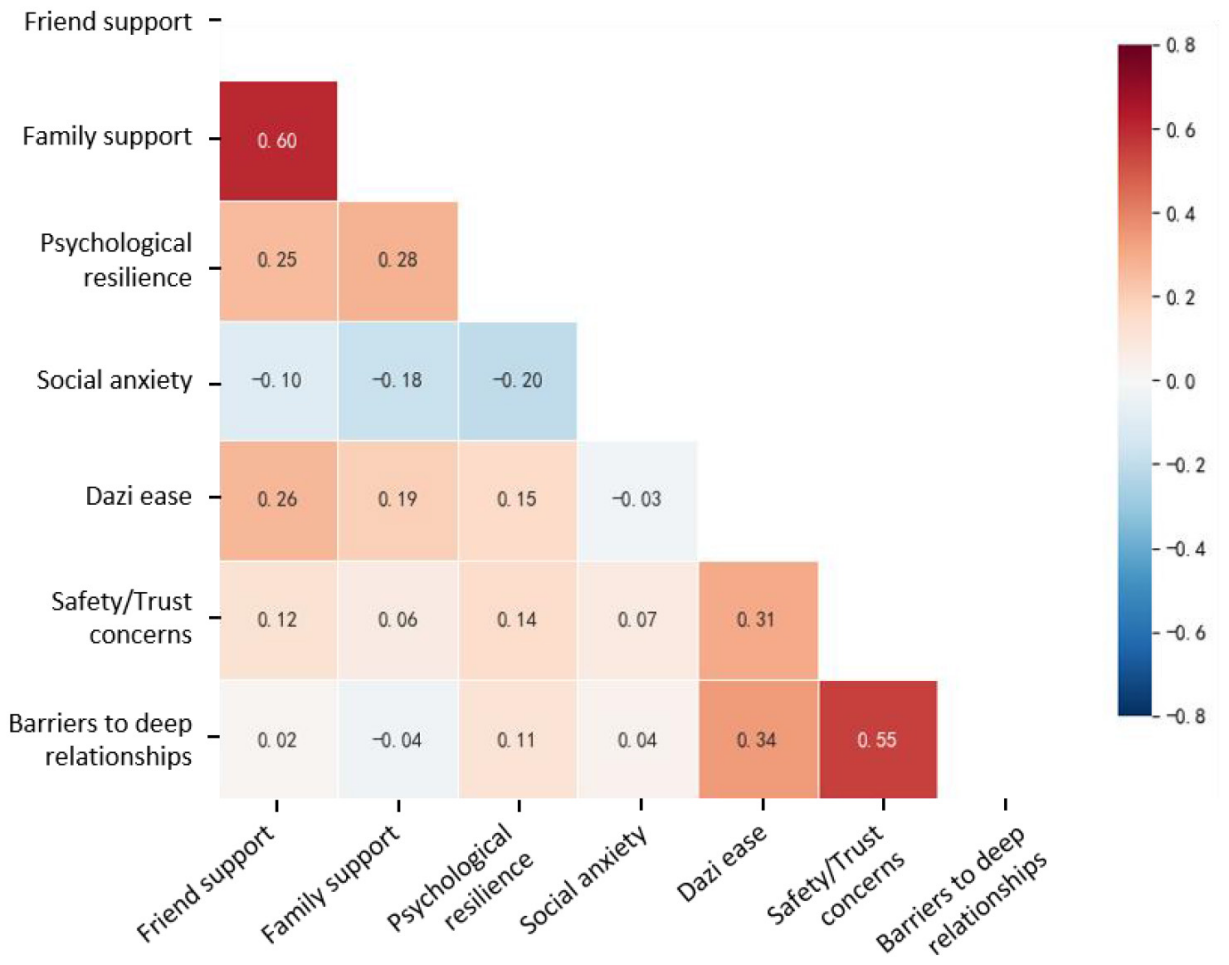
Correlation matrix of core variables within the dazi group (*n* = 362). Friend support = total score of friend support; Family support = total score of family support; Psychological resilience = total score of psychological resilience; Social anxiety = total score of social anxiety; Dazi ease = “When interacting with *dazi*, I feel more relaxed and experience less pressure than when interacting with friends” (rated on a 1–5 scale); Safety/Trust concerns = “I am concerned about encountering safety or trust issues in interactions with *dazi*” (rated on a 1–5 scale); Barriers to deep relationships = “*Dazi* relationships make me feel that establishing deep intimate relationships has become more difficult or that I have become more distant from others” (rated on a 1–5 scale). The matrix presents only the lower triangular portion, and “—” on the diagonal indicates that the correlation coefficient of each variable with itself is 1.00.

Notably, *dazi* ease (i.e., feeling more relaxed and experiencing less pressure when interacting with *dazi* than with friends) was moderately and positively correlated with friend support (*r* = 0.47, *p* < 0.001) and family support (*r* = 0.42, *p* < 0.001), (*r* = 0.15, *p* < 0.01) and social anxiety (*r* = −0.03, *p* = 0.558). *Dazi* ease may represent more of an immediate subjective experience rather than a long-term psychological resource. Although interactions with *dazi* may feel more relaxed and less stressful, such experiences may not be sufficiently associated with lower levels of anxiety. The factor that truly contributes to anxiety reduction may still be the deep, stable, and secure attachment provided by family support.

Furthermore, “Safety/Trust concerns” were highly positively correlated with “Barriers to deep relationships” (*r* = 0.55, *p* < 0.001), indicating that concerns about the safety of *dazi* relationships and fears that such relationships may undermine deeper relationships tend to co-vary substantially, potentially constituting a latent “risk factor of *dazi* social interaction.”

#### 3.5.2 Hierarchical regression: predictive effects of traditional support and *dazi* socializing on social anxiety

To compare the relative contributions of traditional social support and *dazi* socializing to social anxiety, hierarchical regression analysis was conducted (see Table 6). Model 1 included only friend support and family support, *R*^2^ = 0.033 (*F* = 6.10, *p* = 0.003). Among these predictors, family support was a significant predictor of social anxiety (β = −0.157, *p* = 0.012), whereas the independent contribution of friend support was not significant (β = 0.018, *p* = 0.770).

**Table 6:** Hierarchical regression of *dazi* socializing in predicting social anxiety (*n* = 362).

Step	Predictor	β	*t*	*p*	*R* ^2^	Δ*R*^2^
Step 1					0.033	—
	Friend support total score	0.018	0.29	0.770		
	Family support total score	−0.157	−2.52	0.012		
Step 2					0.041	0.008
	Friend support total score	0.018	0.29	0.769		
	Family support total score	−0.157	−2.53	0.012		
	*dazi* enjoyment	−0.207	−1.80	0.073		
	*dazi* ease	−0.071	−0.89	0.376		
	Safety/Trust concerns	0.338	2.64	0.009		

The dependent variable was social anxiety; β represents the unstandardized regression coefficient; Δ*R*^2^ represents the increase in *R*^2^ from Step 1 to Step 2, with F change = 0.98, *p* = 0.403.

Model 2 further incorporated three *dazi* socializing indicators (*dazi* enjoyment, *dazi* ease, and Safety/Trust concerns) based on Model 1. The explained variance increased to *R*^2^ = 0.041, with an increase of Δ*R*^2^ = 0.008 (*F* change = 0.98, *p* = 0.403). Overall, the *dazi* experience indicators did not significantly contribute to the incremental variance explained in social anxiety. Compared with traditional social support, *dazi* socializing demonstrated a relatively weaker contribution to social anxiety. Among these indicators, only “Safety/Trust concerns” reached statistical significance (β = 0.338, *p* = 0.009), indicating that higher social anxiety was indirectly associated with greater “Safety/Trust concerns” in *dazi* relationships.

Assumption checks for the hierarchical regression models indicated acceptable residual normality (Shapiro–Wilk ps < 0.001, Q–Q plots showing mild deviations) and homoscedasticity (Breusch–Pagan ps > 0.05). Variance inflation factors (VIFs) were notably elevated for friend support and family support in both models (VIFs > 10), reflecting the theoretical overlap between these two dimensions of social support (*r* = 0.60, *p* < 0.001). Importantly, multicollinearity does not bias the overall model fit (*R*^2^) or its change (Δ*R*^2^), which were the primary focus of this hierarchical regression analysis.

#### 3.5.3 Comparison of *dazi* motivation groups

To further examine the differential effects of *dazi* motivations, participants who endorsed “companionship needs (avoiding loneliness)” but did not endorse “functional needs (cost-sharing or making up the numbers)” were classified into the emotional-oriented *dazi* group (*n* = 61), whereas those with the opposite pattern were classified into the function-oriented *dazi* group (*n* = 76), and the two groups were compared in terms of psychological adaptation.

An independent-samples *t* test with Welch correction showed (see Table 7) that psychological resilience was lower in the emotional-oriented *dazi* group (84.61 ± 10.39) than in the function-oriented *dazi* group (88.46 ± 12.83), with the difference approaching statistical significance (*t* = −1.94, *p* = 0.054). No significant difference in social anxiety was observed between the two groups (*t* = −0.54, *p* = 0.591). These findings suggest that individuals who seek *dazi* relationships primarily to avoid loneliness may have relatively lower psychological resilience, indicating that individuals with weaker psychological resilience may have a greater need for *dazi* relationships to manage feelings of loneliness. However, differences in motivation did not affect the ultimate outcome: as long as individuals had *dazi* relationships, their social anxiety remained at a relatively low level. This provides a micro-level explanation for the finding in the full-sample mediation analysis that the group with *dazi* experiences had higher psychological resilience: *dazi* relationships may not be the cause of increased psychological resilience; rather, individuals with higher psychological resilience may be more capable of developing different types of *dazi* relationships.

**Table 7:** Comparison of psychological adaptation across different *dazi* motivation types (M ± SD).

Variable	Emotional-oriented *dazi* (*n* = 61)	Function-oriented *dazi* (*n* = 76)	*t*	*p*
Psychological resilience	84.61 ± 10.39	88.46 ± 12.83	−1.94	0.054
Social anxiety	47.43 ± 4.06	47.79 ± 3.74	−0.54	0.591

Emotional-oriented *dazi* = endorsed “companionship needs (avoiding loneliness)” but did not endorse “functional needs (cost-sharing/making up the numbers)”; function-oriented *dazi* = endorsed “functional needs” but did not endorse “companionship needs.”

## 4 Discussion

### 4.1 Traditional social support and social anxiety among medical students: the protective effect of strong ties

This study found that both family support and friend support were associated with lower levels of social anxiety among medical students, supporting H1. This finding is consistent with previous research on university students, which has shown that social support is a key protective factor associated with psychological health and well-being, with relatively robust effects across different cultural contexts (Wang et al., 2026). Among medical students, who constitute a high-stress population, the function of traditional strong-tie support may be particularly salient. Previous studies of Chinese medical students have shown significant negative associations between family functioning and social support and symptoms of anxiety and depression, suggesting that emotional connection within the family and fulfillment of belonging needs through friendships may jointly constitute important psychological resources for medical students in coping with academic and social stress (Li et al., 2025). Notably, after controlling for gender, year of study, and student leadership status, the between-group difference in family support changed from non-significant to significant, suggesting that individuals who are more capable of maintaining family relationships may possess stronger emotional regulation abilities, which may extend to social contexts on campus and enable them to benefit similarly when developing new forms of social relationships.

### 4.2 *Dazi* socializing and social anxiety among medical students: the complementary function of weak ties

This study found that medical students with experience of *dazi* socializing reported lower levels of social anxiety, supporting H1. Previous research has shown that everyday interactions with weak ties are significantly associated with positive emotions and a sense of belonging, and that such interactions often involve relatively low levels of emotional demands (Shi, 2024). A classic study of university students found that when individuals interacted with weak ties more frequently than they typically did in daily life, their well-being and sense of belonging increased significantly (Ishiguro, 2023). For medical students, *dazi* socializing may fit particularly well with their high-pressure and fragmented lifestyles through providing context-specific companionship that meets immediate social needs without imposing the pressure of maintaining long-term emotional relationships. However, this finding should be interpreted with caution, as this study operationalized *dazi* socializing as a binary variable (experience vs. no experience) and therefore did not capture key dimensions such as frequency, duration, or relationship quality. Participants without experience of *dazi* socializing may also have obtained alternative forms of social connection through physical activities, participation in student organizations, or interactions with family members. Therefore, the difference between the two groups should be understood as an association between preferences for different social strategies rather than as a binary distinction between the presence and absence of social support.

### 4.3 The mediating mechanism of psychological resilience: from external support to internal resources

This study found that traditional social support, dazi socializing experiences, and social anxiety may all have indirect associations through psychological resilience. Previous research among university students has shown that emotion regulation strategies and psychological resilience have significant protective effects on mental health under stressful conditions, and that psychological resilience may serve as a bridge between external support resources and internal adaptive outcomes (Brites et al., 2024). In this study, friend support and family support had indirect associations with social anxiety through psychological resilience, suggesting that traditional strong-tie support may operate through a mechanism of “deep empowerment”, whereby long-term and stable emotional investment is associated with individuals' psychological resilience. In contrast, *dazi* socializing experiences had both direct and indirect associations with social anxiety when psychological resilience was included in the model, which may reflect the distinctive “immediate companionship” function of weak-tie support. Low-threshold, context-specific companionship may be associated with emotional relief without requiring complete reliance on the conversion of external resources into psychological resources. The conservation of resources theory provides a complementary perspective for understanding this mechanism: social support, as an external resource, may be associated with individuals' ability to maintain psychological balance in the face of social-evaluative threats by promoting the accumulation of psychological resilience as an internal resource (Hobfoll, 1989).

### 4.4 Theoretical contributions and an integrative interpretation

The theoretical contributions of this study are reflected primarily in three aspects. First, by incorporating *dazi* socializing, an emerging form of weak-tie relationships, into the social support framework, this study extends the application of weak-tie theory to contemporary youth mental health. Previous studies have often conceptualized social support as a linear function of strong ties, whereas the present findings suggest that weak-tie support may have psychological protective functions that are independent of strong ties in specific populations and that its underlying mechanisms may differ from those of strong-tie support. Second, the findings highlight the key role of psychological resilience as a “resource converter.” Traditional strong-tie support may be characterized by “deep empowerment” through a full mediation pathway, whereas *dazi* socializing may offer a dual function of “immediate relief and limited empowerment” through a partial mediation pathway. Third, this study proposes a “support-system complementarity hypothesis,” suggesting that traditional social support and *dazi* socializing are not substitute relationships in a zero-sum competition but rather functionally complementary and synergistic forms of support. Individuals with higher levels of traditional social support may not be less inclined to engage in *dazi* socializing; instead, they may be better able to make use of *dazi* relationships because of their stronger social capabilities. Meanwhile, the immediate companionship associated with *dazi* socializing may partially compensate for the “support vacuum” created by geographical distance and time constraints in traditional social support.

### 4.5 Limitations and future directions

Several limitations of this study should be addressed in future research. First, this study used cross-sectional data and, although the mediation model was constructed based on theoretical assumptions, it cannot establish causal relationships among the variables. Reverse pathways, such as individuals with lower social anxiety being more likely to establish *dazi* relationships, or bidirectional associations may also exist. Future studies could employ longitudinal designs or experimental designs to clarify the direction of causality. Second, the sample was drawn from a single academic discipline and consisted predominantly of first-year students and female students with *dazi* experiences, resulting in a degree of structural imbalance. The social patterns of medical students may differ from those of students in other academic disciplines, and caution should therefore be exercised when generalizing the findings to other populations. Third, all variables in this study were assessed through self-report measures, which may be subject to social desirability effects and common method bias. Future research could incorporate multiple measurement approaches, such as behavioral experiments, peer reports, or physiological indicators. Fourth, this study examined only the mediating role of psychological resilience and did not include other potential mediators or moderators, such as self-esteem, social skills, and personality traits. A single-pathway model may therefore be insufficient to fully capture the complexity of the relationships among the variables. In addition, the relatively small effect sizes may indicate that *dazi* socializing represents only one of many factors associated with social anxiety and psychological resilience, and future research could examine these associations in conjunction with personality traits and other relevant variables.

Additionally, the heterogeneity of regression slopes in the ANCOVA models for friend support, family support, and social anxiety suggests that the relationships between covariates (gender, academic year, and student leadership status) and these outcomes differ somewhat between the *dazi* and non-*dazi* groups. This is not unexpected, given that gender and academic year are precisely the demographic variables most strongly associated with *dazi* experience. Nevertheless, the finding suggests that *dazi* experience itself may interact with demographic variables in shaping psychological outcomes. Future research employing interaction or subgroup analyses could more precisely delineate these moderated relationships. Additionally, the elevated variance inflation factors (VIFs) observed for friend and family support in the hierarchical regression models are theoretically expected given their shared conceptual underpinning. Although multicollinearity does not bias overall model fit, it reduces the precision of individual coefficient estimates; future studies may use structural equation modeling or principal component analysis to more cleanly disentangle the unique contributions of different support dimensions.

### 4.6 Practical implications

Based on the above findings, this study proposes practical recommendations for four groups of stakeholders: mental health education professionals in medical schools, counselors and clinical instructors, and medical students themselves.

For mental health education professionals, *dazi* socializing should be incorporated into the spectrum of social support among young people, with particular attention paid during mental health screening to students who seek *dazi* relationships primarily to avoid loneliness. Dual-track group counseling that integrates social skills and psychological resilience should be developed, with priority given to repairing deficits in traditional support networks while simultaneously fostering the understanding that *dazi* relationships are complementary rather than substitutive.

For counselors and clinical instructors, structured *dazi* matching, such as peer study-support groups, should be facilitated during the first-year adjustment period and transitions into clinical internships to support students in navigating social barriers. Counselors and clinical instructors should also understand the development of students' social networks through dormitory visits, individual conversations, and other forms of communication. At the class level, low-pressure and non-mandatory group activities should be organized to foster light social settings resembling *dazi* relationships.

For medical students themselves, awareness of the need for a diversified support system should be fostered, with active efforts to maintain deep connections with family members and long-term friends while avoiding the use of *dazi* relationships as a means of avoiding deeper emotional investment. Students should exercise caution when using platforms that match them with strangers and should establish clear boundaries in their interactions. They should also make effective use of functional relationships such as “study partners,” integrating social interaction into their academic routines to achieve both social and functional benefits.

## Data Availability

The raw data supporting the conclusions of this article will be made available by the authors, without undue reservation.
